# Identification of Compound CB-2 as a Novel Late-Stage Autophagy Inhibitor Exhibits Inhibitory Potency against A549 Cells

**DOI:** 10.3390/life11080865

**Published:** 2021-08-23

**Authors:** Zhihui Liu, Lu Zhang, Yachao Liu, Hanxiao Zhang, Jingxuan Chen, Gaoqing Feng, Peichang Yang, Fangfang Sha, Liuqing Cui, Gangchun Sun

**Affiliations:** 1 College of Bioengineering, Henan University of Technology, Lianhua Street, Zhengzhou 450001, China; molecule@stu.haut.edu.cn (Z.L.); liuych5@163.com (Y.L.); zhxzhx131@163.com (H.Z.); 201992170@stu.haut.edu.cn (J.C.); 201992160@stu.haut.edu.cn (G.F.); 2020920262@stu.haut.edu.cn (P.Y.); 2020920255@stu.haut.edu.cn (F.S.); cuilq1982@163.com (L.C.); 2 College of Chemistry and Chemical Engineering, Henan University of Technology, Lianhua Street, Zhengzhou 450001, China; sungangchun@163.com

**Keywords:** non-small cell lung cancer, autophagy inhibitor, (E)-3-((E)-4-chlorobenzylidene)-5-((5-methoxy-1H-indol-3-yl)methylene)-1-methylpiperidin-4-one, reactive oxygen species

## Abstract

Autophagy has been recognized as a stress tolerance mechanism that maintains cell viability, which contributes to tumor progression, dormancy, and treatment resistance. The inhibition of autophagy in cancer has the potential to improve the therapeutic efficacy. It is therefore of great significance to search for new autophagy inhibitors. In the present study, after screening a series of curcumin derivatives synthesized in our laboratory, (E)-3-((E)-4-chlorobenzylidene)-5-((5-methoxy-1H-indol-3-yl)methylene)-1-methylpiperidin-4-one (CB-2) was selected as a candidate for further study. We found that CB-2 increased the LC3B-II and SQSTM1 levels associated with the accumulation of autophagosomes in non-small cell lung cancer (NSCLC) A549 cells. The increased level of LC3B-II induced by CB-2 was neither eliminated when autophagy initiation was suppressed by wortmannin nor further increased when autophagosome degradation was inhibited by chloroquine (CQ). CB-2 enhanced the accumulation of LC3B-II under starvation conditions. Further studies revealed that CB-2 did not affect the levels of the key proteins involved in autophagy induction but significantly blocked the fusion of autophagosomes with lysosomes. High-dose CB-2 induced the apoptosis and necrosis of A549 cells, while a lower dose of CB-2 mainly impaired the migrative capacity of A549 cells, which only slightly induced cell apoptosis. CB-2 increased the levels of mitochondrial-derived reactive oxygen species (ROS) while decreasing the mitochondrial membrane potential (MMP). Scavenging ROS via N-acetylcysteine (NAC) reversed CB-2-induced autophagy inhibition and its inhibitory effect against A549 cells. In conclusion, CB-2 serves as a new late-stage autophagy inhibitor, which has a strong inhibitory potency against A549 cells.

## 1. Introduction

Autophagy represents the degradation event with a high evolutionary conservation degree for protein and organelle turnover through lysosomal-dependent degradation pathways [1]. In addition to its essential role in maintaining the balance of the intracellular environment in normal cells, autophagy is involved in various pathological processes, especially cancer [2]. Unlike normal cells, most cancer cells are exposed to hypoxic conditions within tumor regions, and thus, they have elevated levels of basal autophagy to resist environmental stress [3]. The deletion of autophagy-related genes significantly reduces tumor growth in the engineered mouse models for cancer, indicating that autophagy is indispensable for tumorigenesis [4]. Most chemo/radiotherapy inevitably leads to cellular stress. In this case, autophagy is often activated, enabling cancer cells to resist anticancer treatments and facilitating tumor dormancy [5]. The blockade of autophagy forces the proliferation of dormant tumor cells, which makes them more vulnerable to be killed by chemo/radiotherapy [5]. Excitingly, mounting preclinical evidence suggests that the inhibition of autophagy can improve the clinical outcomes of patients suffering from various cancers [6]. Therefore, the inhibition of autophagy emerges as the efficient antitumor treatment.

Currently, chloroquine (CQ), along with the corresponding derivative hydroxyl CQ (HCQ), can be used as the autophagy inhibitors with clinical availability. Studies have shown that using CQ and HCQ monotherapies or in combination is feasible and beneficial for treating diverse malignancies, including non-small cell lung cancer (NSCLC) [7,8,9]. Unfortunately, their lack of potency in modulating autophagy and off-target toxicity greatly limits their further clinical applications [10]. It is therefore of great significance to develop new compounds inhibiting autophagy. 

Natural products (NPs) and their derivatives are increasingly being considered as lead compounds in pharmaceutical chemistry and drug discovery. Approximately 80% of the approved chemotherapeutic drugs in cancer treatments are NPs or their derivatives by structural modifications [11]. The recently discovered novel autophagy inhibitors, such as madangamine A, CA-5f, berberine, elaiophylin, oblongifolin C, etc., are all NPs or directly derived from NPs [12,13,14,15,16]. Curcumin, the *Curcuma longa*-extracted natural polyphenol, can suppress cancer cell growth through inducing excessive autophagy induction and/or impaired autophagic flux [17,18,19]. Although there is abundant evidence of the efficacy and safety of curcumin as an autophagy modulator, its low bioavailability in humans severely restricts its clinical application [20]. However, curcumin can represent a good starting point suitable for further optimization to look for novel autophagy regulators, as a growing number of curcumin derivatives have been discovered with improved anticancer efficacy against NSCLC by impairing autophagy [21]. For example, hydrazinobenzoylcurcumin (HBC) and bisdemethoxycurcumin (BDMC), two curcumin derivatives, induced the accumulation of autophagy marker LC3B-II and killed A549 cells by regulating autophagy [22,23]. Similarly, the curcumin analog tetrahydrocurcumin (THC) displayed potent antiproliferative activity against A549 cells. Further studies have revealed that THC increased the level of Beclin 1, a key protein involved in the induction of autophagy, suggesting that THC regulates autophagy in A549 cells [24]. Our recent study identified a curcumin derivative CA-5f as a novel autophagy inhibitor with a potent anticancer effect against NSCLC [13]. 

**Figure 1:**
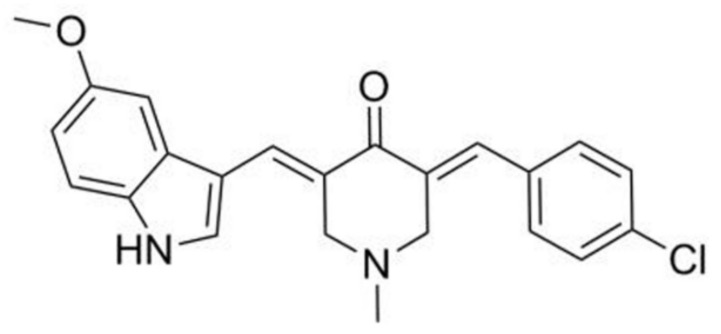
The structure of CB-2. CB-2′s molecular formula and molecular weight are C_23_H_21_ClN_2_O_2_ and 392.88 g/mol, respectively.

This study selected various curcumin derivatives prepared at our laboratory for discovering novel autophagy-targeting inhibitors. Of those derivatives tested, we found that (E)-3-((E)-4-chlorobenzylidene)-5-((5-methoxy-1H-indol-3-yl)methylene)-1-methylpiperidin-4-one (CB-2, Figure 1) potently inhibits the degradation of autophagosomes by blocking their fusion with lysosomes. More excitingly, CB-2 exerts significantly anti-migrative effects against NSCLC A549 cells. Mechanistically, the inhibitory effects of CB-2 on autophagy and its inhibitory potency against A549 cells are because of mitochondrial reactive oxygen species (ROS) overproduction. This work first identifies CB-2 as the new late autophagy-targeting inhibitor, which potently resists NSCLC.

## 2. Materials and Methods

### 2.1. Chemicals and Reagents

The curcumin derivatives, including CB-2, were synthesized in our laboratory. The high-performance liquid chromatography (HPLC) purity of these compounds was at least 99%. Dimethyl sulfoxide (DMSO) was used to prepare the 0.1-M CB-2 stock solution, followed by preservation at −20 °C and dilution to diverse concentrations using a suitable medium prior to utilization. Fetal bovine serum (FBS) was acquired from Hyclone Laboratories (Logan, UT, USA). Both DMEM and RPMI-1640 medium were provided by Gibco, Life Technologies (Carlsbad, CA, USA). Wortmannin (Wort, S1952), DCFH-DA (S0033), N-acetylcysteine (NAC; S0077), and JC-1 (C2006) were provided by Beyotime Biotechnology (Shanghai, China). An Annexin V-FITC/PI apoptosis detection kit (556547) was acquired from BD-Pharmingen (Franklin Lakes, NJ, USA). Earle’s Balanced Salt Solution (EBSS; E2888), thiazolyl blue tetrazolium bromide (MTT; M2128), Protease Inhibitor Cocktail (P8340), and diphenyleneiodonium chloride (DPI; D2926) were provided by Sigma-Aldrich (St. Louis, MO, USA). Paraformaldehyde (E672002-0500) was obtained from Shanghai Sangon Biotech (Shanghai, China). The lactate dehydrogenase detection kit was purchased from the JianCheng Bioengineering Institute (Nanjing, China). 

### 2.2. Antibodies

The primary antibodies anti-LC3B (L7543), anti-SQSTM1 (P0067), anti-phosphor (p)-mTOR (SAB4504476), anti-mTOR (T2949), anti-PIK3C3 (V9764), and anti-β-actin (A5441) were provided by Sigma-Aldrich (St. Louis, MO, USA), while anti-ATG5 (D5F5U), anti-P70S6K (9202S), and anti-p-P70S6K (9206S) were provided by Cell Signaling Technology (Boston, MA, USA), and anti-LAMP1 (21997-1-AP) was provided by Proteintech (Chicago, IL, USA). Meanwhile, the TRITC-labeled goat anti-rabbit IgG (H + L) secondary antibody (ZF-0316) was provided by ZSGB-BIO (Beijing, China), whereas the IRDye^®^ 800CW Goat anti-Mouse IgG (H + L) (925-32210), IRDye^®^ 680RD Goat anti-Rabbit IgG (H + L) (926-68071), and IRDye^®^ 800CW Goat anti-Rabbit IgG (H + L) (925-32211) secondary antibodies were provided by Li-Cor Biotechnology (Lincoln, NE, USA).

### 2.3. Cell Culture and Treatment

We cultured A549 cells within the RPMI-1640 medium that contained 10% FBS. The HepG2, MCF-7, and NCI-H157 cell lines were cultured within DMEM that contained 10% FBS. Afterwards, we incubated all the above cell lines within an incubator under 5% CO_2_ and 37 °C conditions. All the treatments were performed as described below. (1) The cells were treated with CB-2 at diverse doses or DMSO for diverse periods; (2) the cells were treated using inhibitors ahead of time, followed by treatment with 20-μM CB-2 or DMSO for the different times indicated in the figures.

### 2.4. Western Blotting (WB)

A WB assay was conducted according to the previous instructions [13]. Briefly, the cells were rinsed in ice-cold phosphate-buffered saline (PBS) and then collected by a lysis buffer, which contained 2% SDS, 25-mM Tris-HCl (pH 6.8), 6% glycerol, 1% 2-mercaptoethanol, 2-mM PMSF, 0.02% bromophenol blue, and protease inhibitor cocktail. The proteins per lane were separated by 12% SDS-polyacrylamide gel electrophoresis, and the target proteins were transferred onto nitrocellulose membranes. After being blocked by nonfat milk for 1 h, nitrocellulose membranes were incubated with the corresponding primary antibodies overnight at 4 °C and with the appropriate secondary antibodies for 1 h at 37 °C. The immunoblots were processed and analyzed using the Odyssey CLX Infrared Imaging System (LI-COR Biosciences, Cambridge, UK). The integrated fluorescence intensities of the blots were quantified via Odyssey Application Software.

### 2.5. Quantitative Real-Time PCR (qRT-PCR)

This study performed qRT-PCR for detecting SQSTM1 and LC3B mRNA expression according to the previous description [25]. The primer sequences were synthesized by Shanghai Sangon Biotech. The sequences from 5′ to 3′ are shown below. LC3B (forward): AAACGCATTTGCCATCACAGT, LC3B (reverse): GTGAGGACTTTGGGTGTGGTTC, GAPDH (forward): AATGACCCCTTCATTGAC, GAPDH (reverse): TCCACGACGTACTCAGCGC, SQSTM1 (forward): TACGACTTGTGTAGCGTCTGC, and SQSTM1 (reverse): GTGTCCGTGTTTCACCTTCC.

### 2.6. Transmission Electron Microscopy (TEM)

An ultrastructural analysis of the autophagosome-like structure was performed according to a previously reported study [22]. Briefly, after a treatment with DMSO or 20-μM CB-2 for 24 h, the cells were fixed overnight in 3% glutaraldehyde and in 1% osmic acid for 2 h and then dehydrated using 50%, 70%, 80%, 90%, and 100% alcohol and 100% acetone in a series. After dehydration, the samples were embedded in epoxy resin. We utilized the Ultracut UCT ultramicrotome (Leica Microsystems, Wetzlar, Germany) to cut ultrathin sections and employed the JEM-1230 TEM (JEOL, Tokyo, Japan) for the examination.

### 2.7. Immunofluorescence and Confocal Microscopy

First of all, we grew A549 cells onto the glass coverslips that were added in the 24-well plates. Afterwards, the cells were subjected to 30 min of 4% paraformaldehyde fixation on ice and 30 min of 5% BSA blocking after the treatment. Thereafter, anti-LAMP1 primary antibodies were employed to incubate the cells at 4 °C overnight, and later, TRITC-labeled secondary antibodies were adopted to further incubate the cells for 1 h at 37 °C in the dark. DAPI was adopted for staining the nuclei for 15 min. We employed a FLUOVIEW FV3000 confocal laser-scanning microscope (Olympus, Tokyo, Japan) to observe the cells and take images. The transition from autophagosomes to autolysosomes was indicated by the spot number ratio of autolysosomes (LC3^+^LAMP1^+^) to autophagosomes (LC3^+^).

### 2.8. Real-Time Cell Analyzer Assay (RTCA)

In brief, we first grew A549 cells in 16-well electronic microtiter plates and used CB-2 at diverse doses to treat them. The growth of A549 cells was continuously monitored and recorded using the xCELLigence RTCA S16 System (ACEA Biosciences, Santiago de Chile, CA, USA). RTCA S16 Software was utilized to obtain and analyze the data.

### 2.9. Cell Viability Analysis

This study conducted a MTT assay for measuring the cell viability according to a previous description [13].

### 2.10. Annexin V-PITC/PI Double-Staining Analysis

This study conducted Annexin V-FITC/PI double staining to determine the A549 cell apoptosis by the use of an Annexin V-FITC/PI detection kit following specific protocols. At last, FACSCalibur flow cytometry (Becton Dickinson, Franklin Lakes, NJ, USA) was performed to analyze the fluorescence signals.

### 2.11. Colony Formation Assay

First of all, we planted A549 cells (3 × 10^3^/well) into 6-well plates and treated them with CB-2 (20 or 40 μM) for 12 days (replaced with the fresh medium containing CB-2 every 2 days). At day 12, 4% paraformaldehyde was used to fix the cells, followed by 30 min of incubation using a 0.5% crystal violet solution to visualize the colonies.

### 2.12. LDH Assay

The LDH detection kit was used to determine the changes in the LDH levels after 24 h of CB-2 treatment in line with specific instructions. The FLx800™ Multi-Detection Microplate Reader (Bio-Tek, Burlington, UT, USA) was utilized to measure the sample solution absorbance (OD) value at 340 nm.

### 2.13. Wound-Healing Assay

A linear wound was made onto the cell layer using the sterile pipette tip after the A549 cells grew to close confluency. After rinsing by PBS for removing those scraped cells, 20-μM CB-2 was utilized to treat cells with/without 10-mM NAC for a period of 24 or 48 h. Then, a light microscope was utilized to capture the wound images using the camera. This study adopted ImageJ software (Bethesda, MD, USA) to quantify the wound gap width.

### 2.14. Transwell Assay 

We inoculated A549 cells in the upper floor of the 8-µm pore Transwell chambers (Corning, Corning, NY, USA) and incubated them with serum-free RPMI-1640 medium containing CB-2 or/and NAC. The lower chamber was supplemented with a complete medium. After 24 or 48 h, the migrating cells on the upper chamber bottom were subjected to 30 min of 0.25% crystal violet staining before counting. The images were captured by a light microscope equipped with a camera.

### 2.15. Detection of Intracellular and Mitochondrial ROS Levels

The DCHF-DA or MitoSOX^®^ Red fluorescent probes were adopted to detect the intracellular or mitochondrial ROS contents based on the manufacturer’s protocols, respectively. The fluorescence signal was analyzed using FACSCalibur flow cytometry.

### 2.16. Detection of Mitochondrial Membrane Potential (MMP)

We measured the MMP using a JC-1 fluorescence probe based on the manufacturer’s protocols. The fluorescence microscope was utilized to capture fluorescence images. Then, a fluorescence signal was analyzed using FACSCalibur flow cytometry. The red-to-green fluorescence intensity was calculated to determine the MMP.

### 2.17. Statistical Analysis

All statistical analyses were performed using SPSS (version 17.0, Chicago, IL, USA). All the experiments were performed in duplicate, and the data were presented as the means ± standard error of the mean (S.E.M.). The differences between the control and experimental groups were analyzed by a Student’s unpaired *t*-test (two groups) or one-way variance (ANOVA; more than two groups). * *p* < 0.05 and ** *p* < 0.01 stood for statistical significance. “*n*” in the figure legends represents the biological replicates. 

## 3. Results

### 3.1. Discovery of CB-2 as an Autophagy Modulator

**Figure 2:**
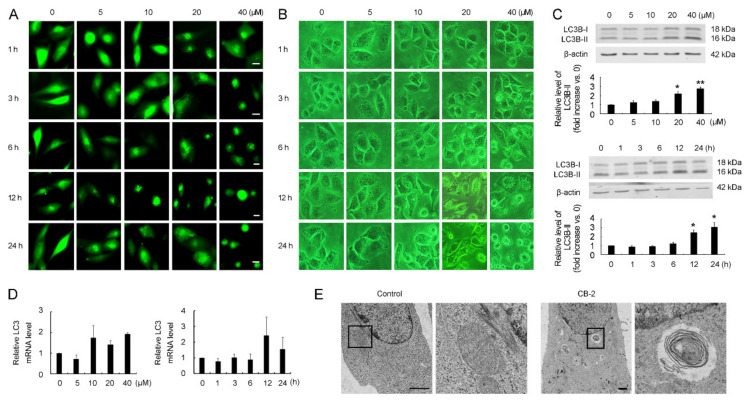
CB-2 regulates autophagy. (**A**) CB-2 induced GFP-LC3B punctate distribution in U87 cells. CB-2 at diverse doses was used to treat U87 cells for diverse periods. Scale bars = 10 μm. (**B**) CB-2 triggered A549 cell morphological alterations. CB-2 at diverse doses was utilized to treat A549 cells for different time points. The magnifications of the images are 400×. (**C**) CB-2 induced LC3B-II accumulation within the A549 cells in a time- and dose-dependent manner. Twenty micrometers of CB-2 or CB-2 at diverse doses were used to treat the cells for diverse time periods or 24 h, respectively. The WB assay was conducted to detect the LC3B-II protein expression. Bar graphs showed the densitometric analysis of the ratio of LC3B-II to β-actin. Data from group 0 were set to 1. (**D**) CB-2 did not affect the mRNA level of LC3B. CB-2 at diverse doses was adopted for 24 h of cell treatment or 20-μM CB-2 for different time points. A qRT-PCR analysis was used for detecting the mRNA level of LC3B. (**E**) CB-2 induced the accumulation of autophagosomes in A549 cells. Twenty micrometers of CB-2 were used to treat the cells for a period of 24 h. An ultrastructural analysis was used for detecting autophagosome-like structures. The boxed areas are enlarged on the right. Scale bars represent 1 μm. *n* = 3; * *p* < 0.05 and ** *p* < 0.01 vs. 0.

A series of derivatives of curcumin were screened for discovering a novel autophagy inhibitor using GFP-LC3B stably transfected U87 cells (a cell line with the LC3B labeled with GFP at its N terminal). Among the derivatives tested, CB-2 (Figure 1) induced the aggregation of GFP-LC3B in a concentration- and time-dependent manner (Figure 2A), suggesting that CB-2 might modulate autophagy. A morphological analysis showed that CB-2 induced cell vacuolation but not detachment from the culture dish at ≤20 μM, a distinguishing feature from 40-μM CB-2 that a higher shrinking cell count was seen from the cells detaching from the dish bottom (Figure 2B). After AO staining, bright red fluorescent dots appeared in A549 cells treated with CB-2 for 24 h, indicating that this compound induced the accumulation of acidic vesicular organelles in the cytoplasm (Figure S1). In addition, CB-2 at 40 μM triggered great apoptosis-related nuclear alterations, such as nuclear fragmentation and chromatin condensation (Figure S1). The WB analysis indicated that CB-2 promoted LC3B-II expression depending on the time and dose (Figure 2C). Such a consequence was not the result from the boosted transcription, since the LC3B mRNA levels were not enhanced upon the CB-2 treatment (Figure 2D). The ultrastructural investigation showed that, unlike the untreated cells, autophagosomes with a characteristic double- or multimembrane structure were easily observed in CB-2-treated A549 cells (Figure 2E). Based on these results, CB-2 was chosen as a hit compound for further investigation.

### 3.2. Identification of CB-2 as a Novel Inhibitor Targeting Autophagy at a Late Stage

**Figure 3:**
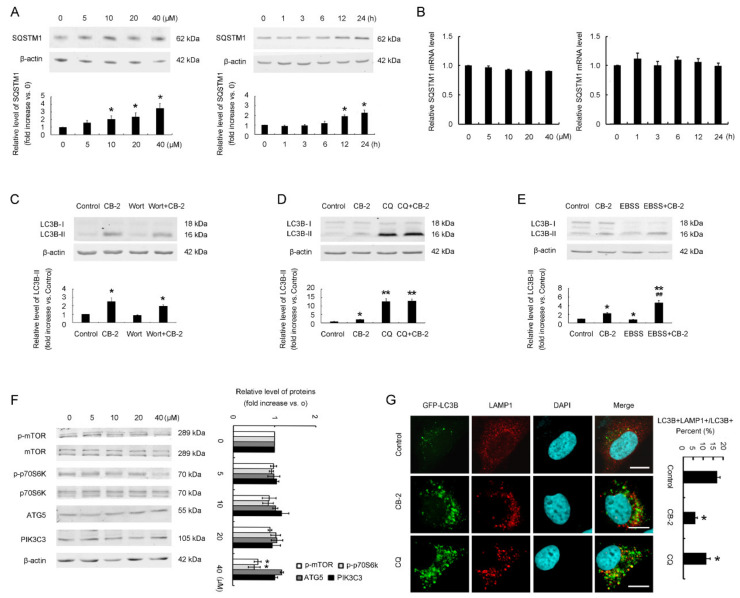
CB-2 serves as the new late-autophagy-targeting inhibitor. (**A**) CB-2 caused SQSTM1 accumulation within A549 cells in a concentration- and time-dependent manner. Twenty micrometers of CB-2 or CB-2 at diverse doses were used to treat cells for diverse periods or for 24 h. A WB assay was adopted for detecting the SQSTM1 protein expression. Bar graphs showed the densitometric analysis of the ratio of SQSTM1 to β-actin. The data from group 0 were set to 1. (**B**) CB-2 did not affect the mRNA level of SQSTM1. Twenty micrometers of CB-2 or CB-2 at diverse doses were used to treat cells for diverse periods or for 24 h. A qRT-PCR analysis was used for detecting the mRNA level of SQSTM1. (**C**) The early-stage autophagy inhibitor, wortmannin (wort), made no difference to the CB-2-induced LC3B-II accumulation. Twenty micrometers of CB-2 were used to treat the A549 cells for 24 h with/without 2-μM wort. A WB assay was conducted to detect the LC3B-II protein expression. Bar graphs showed the densitometric analysis of the ratio of LC3B-II to β-actin. The data from the control group were set to 1. (**D**) The cotreatment of the A549 cells with CB-2 and CQ (inhibitor of late-stage autophagy) made no difference to the LC3B-II accumulation in comparison with the cells exposed to the CQ treatment only. The A549 cells were exposed to 20-μM CB-2 for 24 h with/without 30-μM CQ. A WB analysis was conducted to detect the LC3B-II protein expression. Bar graphs showed the densitometric analysis of the ratio of LC3B-II to β-actin. The data from the control group were set to 1. (**E**) CB-2 promoted LC3B-II accumulation in the absence of nutrients. Twenty micrometers of CB-2 were used to treat the A549 cells within EBSS or the complete medium for 24 h. A WB analysis was conducted for detecting the LC3B-II protein expression. Bar graphs showed the densitometric analysis of the ratio of LC3B-II to β-actin. The data from the control group were set to 1. (**F**) CB-2 did not affect the protein level of several key regulators associated with autophagy induction. The cells were exposed to CB-2 at diverse doses for a period of 24 h; then, a WB assay was conducted to detect the p-p70S6K, p-mTOR, PIK3C3, and ATG5 protein expressions. Bar graphs showed the densitometric analysis of the ratio of p-mTOR to mTOR, p-p70S6K to p70S6K, and ATG5 or PIK3C3 to β-actin. The data from the control group were set to 1. (**G**) CB-2 prevented autophagosomes from fusing with lysosomes. The cells were exposed to 20-μM CB-2 or 30-μM CQ for 24 h. Autophagosome marker GFP-LC3B and lysosome marker LAMP1 colocalization was detected through an immunofluorescence analysis. Scale bars = 10 μm. The histogram showed the autolysosome percentage (LAMP1^+^LC3B^+^, yellow) relative to the total autophagic vesicles (LC3B^+^, green). *n* = 3; * *p* < 0.05 and ** *p* < 0.01 versus 0 or the control; ## *p* < 0.01 versus EBSS.

CB-2 induced an accumulation of autophagosomes, was evidenced through an increased aggregation of GFP-LC3B, enhanced the LC3B-II levels, and increased the number of double- or multimembrane vesicles, due to either excessive autophagy induction and/or impaired autophagosome degradation. To discriminate between these two possibilities, we first analyzed the SQSTM1/p62 levels in CB-2-treated A549 cells. SQSTM1 serves as an adaptor for delivering ubiquitinated proteins into autophagosomes and is degraded along with the complete autophagic process [26]. Thus, the accumulation of SQSTM1 is frequently used as a sign of autophagic flux impairment. The Western blot analysis indicated that CB-2 enhanced the SQSTM1 expression depending on the time and dose (Figure 3A). Such a consequence did not result from the boosted transcription, since the SQSTM1 mRNA levels were not affected by the CB-2 treatment (Figure 3B). After the CB-2 treatment, SQSTM1 and LC3B-II accumulated in NCI-H157 NSCLC cells and in other tumor cell types, including human breast cancer (BC) MCF-7 cells and hepatocellular carcinoma (HCC) HepG2 cells (Figure S2). Next, we performed autophagic flux assays by treating the A549 cells with wortmannin (wort), which inhibits early-stage autophagy through inhibiting the formation of autophagosomes, or chloroquine (CQ), which suppresses late-stage autophagy through preventing autophagosome–lysosome fusion. As revealed by the WB analysis, the CB-2-induced accumulation of LC3B-II was not eliminated when the autophagy initiation was suppressed by wort (Figure 3C). A CQ co-stimulation promoted LC3B-II expression upon the occurrence of autophagy. We found that a cotreatment with CB-2 and CQ did not increase the LC3B-II expression relative to the CQ exposure (Figure 3D), excluding the possibility that CB-2 induces autophagy in A549 cells. Besides, CB-2 further promoted LC3B-II upregulation within the A549 cells in the absence of nutrients in comparison with those cells grown in starvation conditions (Figure 3E). The above findings collectively suggested that CB-2 inhibits autophagosome degradation instead of promoting its synthesis.

Moreover, according to the WB analysis, the expression of several key regulators associated with autophagy induction, including autophagy-related gene 5 (ATG5), p-p70 ribosomal protein S6 kinase (p-p70S6K), phosphorylated mechanistic target of rapamycin kinase (p-mTOR), and phosphatidylinositol 3-kinase catalytic subunit type 3 (PIK3C3), were not altered in the CB-2-treated cells (Figure 3F), supporting that CB-2 affects autophagosome degradation instead of its synthesis.

The immunofluorescence analysis revealed that the colocalization between the LC3B-positive autophagosomes and LAMP1-positive lysosomes in CB-2-exposed cells markedly decreased in comparison with the untreated cells (Figure 3G), suggesting that CB-2 inhibits autophagosome degradation by blocking autophagosome–lysosome fusion.

### 3.3. CB-2 Exhibits Inhibitory Activities against A549 Cells

**Figure 4:**
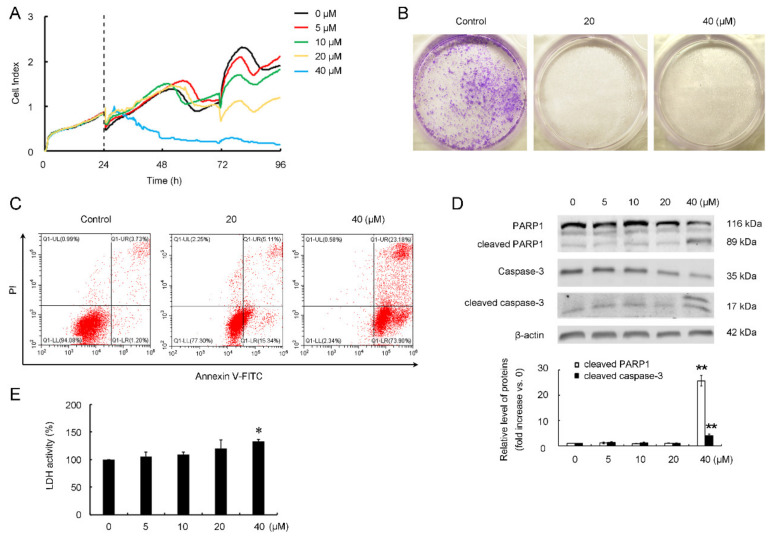
CB-2 exhibits an inhibitory potency against A549 cells. (**A**) CB-2 suppressed the cell viability of A549 cells. CB-2 at diverse doses was used to treat cells; then, the xCELLigence RTCA S16 System was utilized to monitor the cell viability in a real-time manner. The vertical dotted line indicates the CB-2 addition time points. (**B**) CB-2 suppressed the growth of A549 cells. After 12 days of cell culture by 20/40-μM CB-2, a colony formation assay was conducted to determine the cell growth. (**C**) High-dose CB-2 induced the significant apoptosis of A549 cells, while lower-dose CB-2 only slightly induced the early-stage apoptosis of A549 cells. After 24 h of cell culture by 20/40-μM CB-2, the cells were stained using Annexin V-FITC/PI. A cytometric analysis was conducted for determining the cell apoptosis. (**D**) High-dose CB-2 increased caspase-3 and PARP1 cleavage. CB-2 at diverse doses was utilized to treat cells for a period of 24 h; then, a WB assay was conducted to detect cleaved caspase-3 and PARP1 protein expression. Bar graphs showed the densitometric analysis of the ratio of cleaved PARP1 to PARP1 or cleaved caspase-3 to caspase-3. The data from the control group were set to 1. (**E**) High-dose CB-2 induced necrosis of the A549 cells. CB-2 at diverse doses was used to treat the cells for a period of 24 h, and later, a LDH assay was conducted to measure the necrosis. *n* = 3; * *p* < 0.05 and ** *p* < 0.01 versus 0.

To investigate whether CB-2 has inhibitory activities against A549 cells, we first examined how CB-2 affected A549 cell growth by a cell analysis in a real-time manner. CB-2 at low doses of 5 or 10 μM induced no obvious cell number reduction (Figure 4A). At 48-h post-treatment with 20-μM CB-2, the number of A549 cells decreased; since then, such a trend has been continued (Figure 4A). When exposed to 40-μM CB-2, the number of A549 cells displayed a quick decrease within 24 h (Figure 4A), suggesting that CB-2 at 40 μM is highly lethal to A549 cells. A colony formation assay, another way to measure the cytotoxicity of CB-2 to A549 cells, was performed and obtained similar results. Startlingly, CB-2 totally suppressed A549 cell colony formation at the concentrations of 20 or 40 μM (Figure 4B). The results of the MTT assays showed that CB-2 at 20 μM led to a decrease in cell viability of nutrient-starved A549 cells (Figure S3).

Next, flow cytometry was conducted to determine how CB-2 affected A549 cell apoptosis through Annexin V/PI double staining. Relative to the untreated cells, CB-2 at a dose of 20 μM enhanced the proportion of Annexin V-positive cells from 5.40% ± 0.39% to 19.67% ± 2.43%, while CB-2 at the high dose of 40 μM increased the proportion of Annexin V-positive cells to 87.20% ± 7.18% (Figure 4C). A Western blot analysis indicated that CB-2 at doses equal to or below 20 μM did not affect the cleaved PARP1 or caspase-3 levels. By contrast, after the treatment with 40-μM CB-2, the cleaved PARP1 and caspase-3 levels were markedly enhanced within the A549 cells (Figure 4D). Additionally, CB-2 at 40 μM caused LDH release (Figure 4E), indicating that high-dose CB-2 caused apoptosis and necrosis, but lower-dose CB-2 only slightly induced early-stage apoptosis in A549 cells.

### 3.4. CB-2 Inhibits Migration of A549 Cells

**Figure 5:**
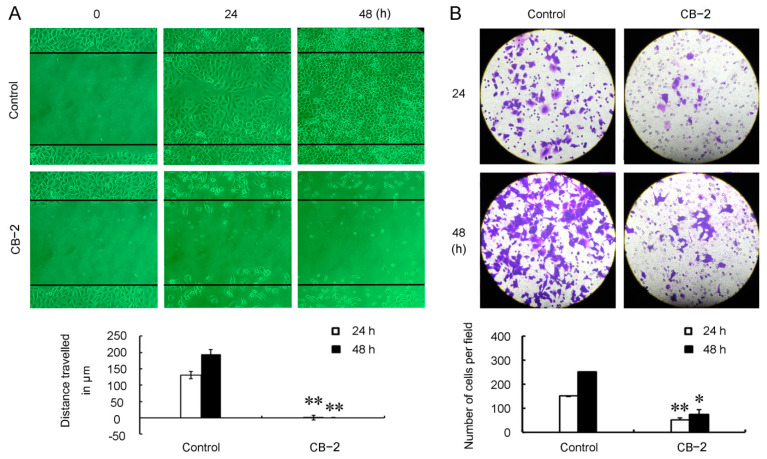
CB-2 impairs the migrative capacity of A549 cells. (**A**) A wound-healing experiment was conducted for determining the cell migration. A histogram displayed the A549 cell relative migrating distance. (**B**) A Transwell migration experiment was adopted for measuring the cell migration. The histogram displays the migrating A549 cell number. *n* = 3; * *p* < 0.05 and ** *p* < 0.01 versus the control).

Considering that a low dose of CB-2 did not cause a large number of cell apoptosis, but it almost completely inhibited the colony formation of A549 cells, a phenotype similar to those resulted from a high dose of the CB-2 treatment. We next determined whether a low dose of CB-2 affects the migrative capacity of A549 cells. A scratch wound-healing assay showed that the A549 cells in the control group gradually migrated into the denuded area and closed the scratch within 48 h. At 48 h post-treatment with 20-μM CB-2, the inhibition rate of A549 cell migration was almost 100% (Figure 5A). In addition, we performed the Transwell chamber assay to further confirm the anti-migrative activity of CB-2 against A549 cells. In agreement, the number of cells treated with 20-μM CB-2 penetrating the membrane in the Transwell assay was much lower than that of the untreated cells (Figure 5B). These results collectively demonstrated that low-dose CB-2 strongly impaired the migrative capacity of A549 cells.

### 3.5. CB-2 Induces Overproduction of Mitochondrial-Derived ROS and Decrease of MMP

**Figure 6:**
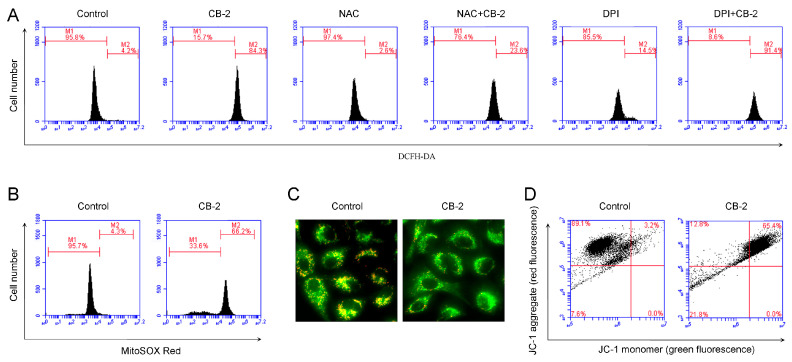
CB-2 induces the overproduction of mitochondrial-derived ROS and decreased the MMP. Twenty micrometers of CB-2 were used to treat the A549 cells for 3 h with/without 10-mM NAC or 10-μM DPI. (**A**) The ROS scavenger NAC, rather than DPI, scavenged the excessive production of ROS induced by CB-2. A flow cytometric analysis of the production of intracellular ROS using DCHF-DA fluorescent probes. (**B**) CB-2 induced the overproduction of mitochondrial-derived ROS. Mitochondrial-derived ROS were monitored by flow cytometry using MitoSOX Red fluorescent probes. (**C,D**) CB-2 decreased the MMP in the A549 cells. The MMP was detected using JC-1 fluorescent probes. Fluorescence photographs (400×) (**C**) and flow cytometry (**D**) of JC-1 staining. *n* = 3.

The overproduction of ROS has been reported to impair the autophagic flux and subsequentially cause the cell death of various types of cancer cells. Thus, we next examined the changes of intracellular ROS upon the CB-2 treatment. A flow cytometric analysis showed that CB-2 at a concentration of 20 μM remarkably promoted the ROS levels within A549 cells, while they were suppressed after the NAC treatment; on the contrary, diphenylene iodonium (DPI), the NADH/NADPH oxidase (NOXs) specific inhibitor (Figure 6A), had no similar effects on ROS clearance, excluding the possibility that CB-2 enhanced the level of NOXs-derived ROS. Unlike normal cells, cancer cells usually have dysfunctional mitochondria due to frequent fission and fusion events [27]. Dysfunctional mitochondria are considered a major source of excessive ROS generation [28]. Thus, the level of mitochondrial ROS was examined using MitoSOX Red, a probe that selectively detects mitochondrial superoxide. We discovered that the level of mitochondrial ROS in CB-2-treated A549 cells significantly increased relative to the untreated cells (Figure 6B). In addition, this study examined the MMP by JC-1, a mitochondrial probe. Under fluorescence microscopy, the cells treated with 20-μM CB-2 showed an enhanced green fluorescence intensity and decreased red fluorescence intensity as compared to the untreated cells (Figure 6C). Similar results were obtained by the flow cytometric analysis (Figure 6D). These data suggested that CB-2 decreased the MMP and increased the mitochondrial-derived ROS production in A549 cells.

### 3.6. CB-2 Inhibits Autophagy and Exerts Inhibitory Potency against A549 Cells via ROS-Dependent Manner

**Figure 7:**
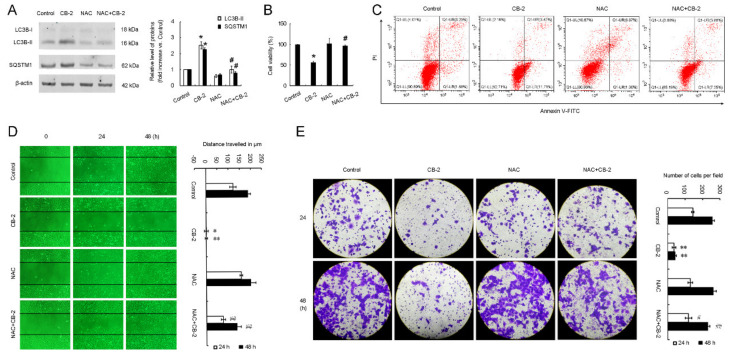
ROS clearance reverses CB-2-induced autophagy inhibition and its inhibitory activity against A549 cells. Twenty micrometers of CB-2 were utilized to treat A549 cells for 24 h (**A**–**E**) or 48 h (**D**,**E**) with/without 10-mM NAC. (**A**) NAC inhibited SQSTM1 and LC3B-II upregulation within CB-2-exposed A549 cells. A WB assay was performed to detect the SQSTM1 and LC3B-II protein expressions. Bar graphs showed the densitometric analysis of the ratio of LC3B-II or SQSTM1 to β-actin. The data from the control group were set to 1. (**B**) NAC reversed the inhibition of CB-2 on the A549 cell viability. We carried out a MTT assay for detecting the cell viability. (**C**) NAC decreased the CB-2-mediated apoptosis. Apoptosis was measured via Annexin V-FITC/PI staining through flow cytometry. (**D**,**E**) NAC reversed the anti-migrative effect of CB-2 on the A549 cells. Scratch (**D**) and Transwell migration (**E**) assays were conducted to determine the cell migration. A histogram displayed the A549 cell relative migrating distance (**D**) and the number of migrative A549 cells (**E**), respectively. *n* = 3; * *p* < 0.05 and ** *p* < 0.01 versus the control; # *p* < 0.05 and ## *p* < 0.01 versus CB-2.

We next investigated whether the overproduction of ROS contributes to CB-2-induced autophagy inhibition and its inhibitory effects on A549 cells. According to the WB results, supplementing NAC totally abolished the SQSTM1 and LC3B-II accumulation caused by CB-2 in A549 cells (Figure 7A). MTT assays showed that NAC reversed the CB-2-induced reduction in the cell viability (Figure 7B). Compared with the cells treated with 20-μM CB-2, a cotreatment with NAC reduced the proportion of Annexin V-positive cells from 19.67% ± 2.43% to 9.53% ± 1.88%, suggesting that ROS are required for CB-2-induced apoptosis in A549 cells (Figure 7C). Furthermore, the wound-healing and Transwell assays indicated that NAC-mediated ROS scavenging nearly abolished the CB-2-induced anti-migration of the A549 cells (Figure 7D,E). The above findings collectively demonstrated that CB-2 inhibits autophagy and exerts inhibitory potency against A549 cells in a ROS-dependent manner.

## 4. Discussion

Autophagy is the degradation process with a high conservation degree within eukaryotes critical for degrading redundant proteins and damaged organelles [29]. Many types of tumor cells increase autophagy to provide a sustainable source of biomolecules and the energy to maintain cell survival under stressful conditions during cancer progression and to resist chemotherapy agents and radiation [30].

In mice with established NSCLC, tumor cells suffered remarkable death and regression after the systematic genetic ablation of autophagy-related gene 7, which indicates the important effect of autophagy on maintaining NSCLC growth and survival [31]. Autophagy has been considered a key factor of the high resistance of NSCLC to chemo/radiotherapy [32,33]. Preclinical studies showed that the inhibition of autophagy inhibited tumor development in mice with established NSCLC [31]. A recent clinical trial reported that the suppression of autophagy by HCQ overcame chemotherapy resistance in advanced NSCLC [7]. Currently, CQ together with the corresponding derivative HCQ are the only approved autophagy inhibitors; however, the long-term administration of CQ or HCQ can result in retinopathies, which limits their clinical use [10]. Thus, the discovery of novel autophagy inhibitors with potential anticancer activities is urgently needed.

During the past decades, NP-based agents have attracted great interest in cancer treatments. Among these NPs, the most abundant and widely studied is curcumin, which delays the development of NSCLC by broadly affecting various molecular targets and signal transduction pathways [34]. Unfortunately, curcumin has a low water solubility, which is still a major challenge in its clinical utilization [34]. Different curcumin derivatives offer promising solutions for overcoming these obstacles and improving the pharmacokinetics of curcumin. This study screened various curcumin derivatives prepared at our laboratory for identifying new autophagy-targeting inhibitors. Among these compounds, we found that CB-2 induced an accumulation of autophagosomes in A549 cells. Other studies demonstrated that CB-2 inhibited late-stage autophagy through suppressing autophagosome–lysosome fusion. The fact that A549 cells exposed to CB-2 exhibited bright red fluorescence in the cytoplasm, which represents an accumulation of acidic compartments, suggests that CB-2 inhibits the degradation of autophagosomes independently of lysosomal alkalization. CB-2 at a high dose or a lower dose significantly inhibited the growth of A549 cells. High-dose CB-2 caused apoptosis and necrosis in A549 cells, while low-dose CB-2 mainly impaired the migrative capacity of the A549 cells, which only slightly induced cell apoptosis. This study identified CB-2 as the new inhibitor for late-stage autophagy that showed inhibitory potency against A549 cells. It is noteworthy that the phenomenon of LC3B-II and SQSTM1 accumulation observed in CB-2-treated A549 cells could also be detected in other cancer cell types, suggesting that CB-2 may have a universal inhibitory effect on autophagy.

Accumulating evidence showed that curcumin derivatives can trigger excessive ROS production in NSCLC cells. For example, L48H37, a curcumin derivative, significantly reduced the growth of A549 and H460 cells through enhanced ROS generation [35]. In another study, Zhao et al. reported that curcumin derivative T59 caused H1975 and A549 cell apoptosis by elevating the ROS level [36]. The present work identified that CB-2 remarkably promoted intracellular ROS generation, which were mainly derived from the dysfunctional mitochondria. The excessive mitochondrial ROS are suggested to exert a vital part in inducing autophagy dysfunction, and this decides the tumor cell fate [37]. Given that CB-2 potentiated mitochondrial ROS generation, it would be interesting to investigate whether excessive ROS induced by CB-2 contributes to suppressing autophagy. According to our results, LC3B-II and STSQM1 accumulation induced by CB-2 was inhibited by a ROS-scavenging agent, NAC. In addition, the inhibitory effects of CB-2 on the growth and migration of A549 cells were substantially reversed by scavenging ROS, clearly suggesting that ROS was important for the activity of CB-2 in inhibiting autophagy and its inhibitory potency against A549 cells.

## 5. Conclusions

In this study, compound CB-2, a curcumin derivative, was determined to be the new inhibitor for late-stage autophagy with an inhibitory potency against NSCLC A549 cells. CB-2 exhibits a concentration effect on the cell fate of A549 cells, i.e., a high dose of CB-2 induces apoptosis and necrosis, while a low dose of CB-2 mainly inhibits migration. CB-2 inhibits autophagy and exerts inhibitory activities against A549 cells by inducing the overproduction of mitochondrial ROS. CB-2 can potentially be established as the novel probe for exploring the autophagosome–lysosome fusion mechanism and a novel therapeutic agent targeting ROS for inhibiting the growth and migration of NSCLC. The anticancer activities of CB-2 in vivo warrant further investigation. Moreover, an in-depth examination of whether the combined use of CB-2 enhances the tumor cell sensitivity to chemo/radiotherapy would be of great significance.

## Data Availability

The data that support the findings of this study are available from the corresponding author upon reasonable request.

## Supplementary Materials

The following are available online at https://www.mdpi.com/article/10.3390/life11080865/s1, Figure S1: CB-2 induces the accumulation of acidic vesicular organelles in the cytoplasm of A549 cells, Figure S2: CB-2 induces the accumulation of LC3B-II and SQSTM1 in another NSCLC cell line H157, the human hepatocellular liver carcinoma cell line HepG2, and human breast carcinoma cell line MCF-7, Figure S3: CB-2 treatment leads to a decrease of cell viability of A549 cells upon starvation stress.
