# Supplementary material for: Identification of Compound CB-2 as a Novel Late-Stage Autophagy Inhibitor Exhibits Inhibitory Potency against A549 Cells
Source: Life (Basel). 2021 Aug 23;11(8):865. doi: 10.3390/life11080865 (PMC8401303; doi:10.3390/life11080865)
Supplement: Supplementary file 1 [file life-11-00865-s001.zip › life-1314753-supplementary.pdf]

*Supplementary Materials*

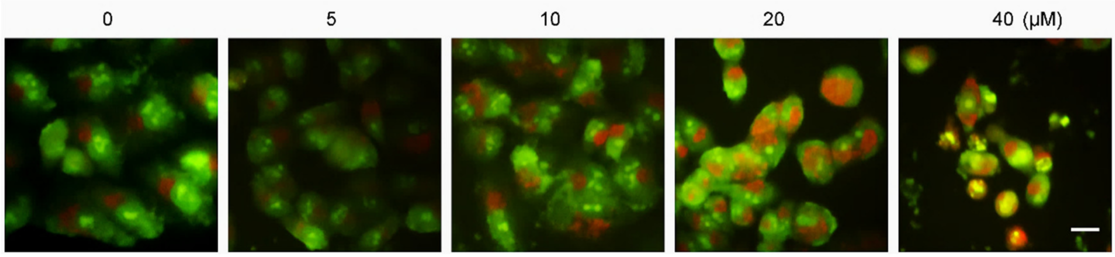


**Figure S1.** CB-2 induces the accumulation of acidic vesicular organelles in the cytoplasm of A549 cells. Fluorescence photographs of acridine orange (AO) staining of A549 cells treated with various doses (0-40 μM) of CB-2 for 24 h. Scale bar represent 10 μm.

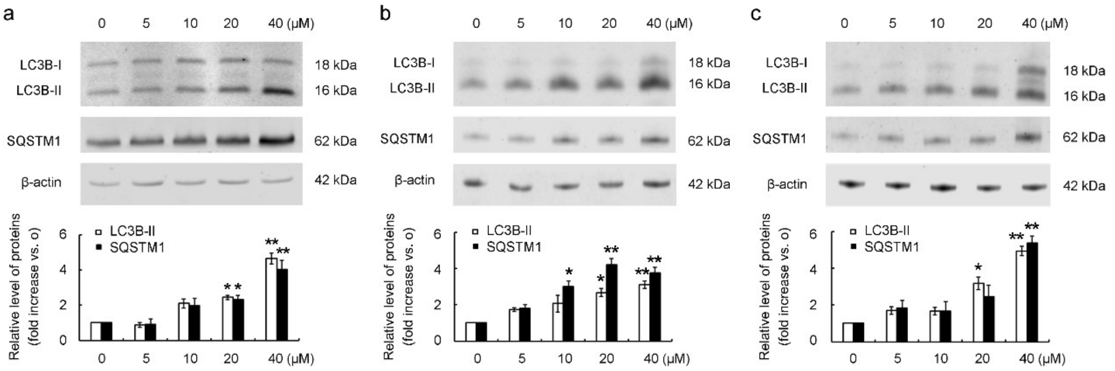


**Figure S2.** CB-2 induces the accumulation of LC3B-II and SQSTM1 in another NSCLC cell line H157 **a**, the human hepatocellular liver carcinoma cell line HepG2 **b,** and human breast carcinoma cell line MCF-7 **c**. Western blot analysis of LC3B-II and SQSTM1 levels in A549 cells treated with various doses (0-40 μM) of CB-2 for 24 h. β-actin was used as a loading control. (n = 3; * $p < 0.05$, ** $p < 0.01$ vs. 0).

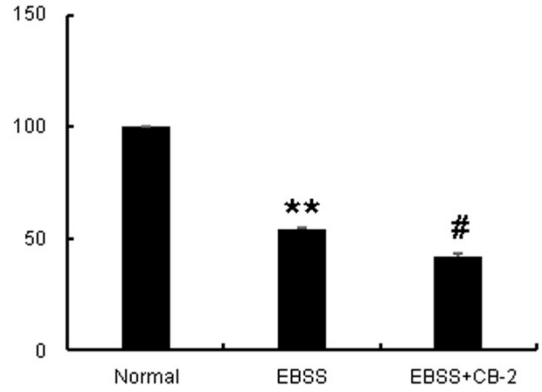


**Figure S3.** CB-2 treatment leads to a decrease of cell viability of A549 cells upon starvation stress. MTT assay for detecting cell viability of A549 cells treated with CB-2 in starvation condition for 24 h. (n = 3; ** $p < 0.01$ vs. Normal, # $p < 0.05$ vs. EBSS).
